# Immigration Policies Shape Latinos’ Trust in Health Information

**DOI:** 10.1007/s40615-025-02566-8

**Published:** 2025-07-23

**Authors:** Sharon Tafolla, Alec M. Chan-Golston, Irene H. Yen, Maria-Elena De Trinidad Young

**Affiliations:** University of California, Merced, School of Social Science Humanities and Arts, 5200 N. Lake Road, Merced, CA 95343 USA

**Keywords:** Immigration policy, Distrust, Latino trust, Immigrant health, Health information, Immigration news coverage

## Abstract

**Context:**

Immigration policy, and especially restrictive policies, are linked to health care access, and can lead to distrust and avoidance of health services. Immigration policy contexts are shaped by media coverage, but we know little about the link between how media covers immigration policy and Latino health care access. We looked at how media covered immigration policy and if it is related to Latinos’ distrust of health information from the government.

**Methods:**

To examine the relationship between media coverage of immigration policy and distrust, we used data from a content analysis of immigration policy news stories in 15 US states between 2010–2013 and 2017–2019 and a multiethnic sample from the Health Information National Trends Survey (HINTS), to assess the relationship between coverage of inclusive and exclusive immigration policies and levels of distrust in health information from the government.

**Findings:**

The results show news coverage of both inclusive and exclusive immigration policies was associated with higher distrust in health information from the government among all respondents. There were no significant differences in the association between high distrust and health information among Latinos, compared to non-Hispanic White and Black population groups.

**Conclusions:**

The paper suggests that immigration as a news topic may also have a role in shaping distrust in health information from the government that is independent of immigration policy. In particular, even coverage of inclusionary immigration policies may not be protective to Latino and immigrant trust in health information as expected.

**Supplementary Information:**

The online version contains supplementary material available at https://doi.org/10.1007/s40615-025-02566-8.

## Introduction

Immigration policies or – related factors, such as restrictive immigration law enforcement, are associated with immigrants’ delay or avoidance of public benefits [1]. Immigration policy-related factors can lead to distrust of government institutions and health care providers among Latino and immigrant populations [2, 3]. News coverage of exclusionary immigration policies and anti-immigrant rhetoric may be an immigration policy-related factor that shapes this distrust. News sources cover exclusionary immigration policies at a high frequency and coverage of exclusionary policies has occurred despite an increase in the enactment of inclusive immigrant policies across several states in the United States [4]. Yet, there has been little research on the role of news coverage in shaping how immigration policies are disseminated and understood to then influence distrust in health information from the government for immigrants.

In this article, we seek to understand the relationship between news coverage of immigration policies, such as those that criminalize or extend benefits for noncitizens, and distrust in health information from the government. We examine news coverage as a “social constructor” of immigration policy, a mechanism by which policy is understood by a community. We use measures from a quantitative content analysis of local newspapers and a multiethnic sample from the Health Information National Trends Survey (HINTS) to test whether the news coverage of immigration policies in a state is associated with high distrust in health information from the government and look for differences in distrust between Latinos and other racial/ethnic groups.

### Immigration Policies and Climates Shaping Latino and Immigrant Health

Immigration policies shape the health and well-being of immigrants directly by shaping access to health care, safety-net benefits, or employment opportunities [5–8]. Moreover, federal and state immigration policies include or exclude immigrants from society based on their legal status [9]. Evidence of the relationship between immigration policies and Latino and immigrant health outcomes show that these policies create conditions or environments that contribute to health inequities [10]. Therefore, not only are *enacted* policies associated with health outcomes, but *proposed* policies, which are often introduced to the public through news or media sources, are also associated with health [11, 12].

Although immigration policy is primarily enacted at the federal level, there are an increasing number of immigrant policies at the state and local level that shape social climates for immigrants. Inclusive immigrant policies may provide immigrants access to public benefits, such as a driver’s license or health care. Inclusive immigrant policies that expand access to these benefits are associated with improved health outcomes for immigrants and their children [13]. On the other hand, exclusionary state immigrant policies shape health by removing or restricting benefit access for immigrants. Targeted enforcement policies or the creation of laws that criminalize immigrants’ use of public benefits can prompt fear of deportation [1, 14]. These exclusionary policies create climates that hinder local efforts to promote the integration of immigrants into a community. For example, in a state that allows or encourages law enforcement to inquire into someone’s immigration status, immigrants may avoid contacting law enforcement due to fears of their legal status being disclosed [3]. Restrictive immigration policy climates are also associated with fewer health care provider visits among immigrant adults [15]. Overall, restrictive immigration policies that target immigrants increase the fear of deportation and can lead to worsened physical and mental health [16].

### Immigration Policies and Anti-immigrant Rhetoric in the News

News coverage reflects the social climates surrounding changes in immigration policy and waves of anti-immigrant rhetoric. There is an opportunity to further understand immigration policy as a social determinant of health by assessing if news coverage of immigration policies contributes to fear of seeking or avoidance of health care and other resources [10]. For example, the media widely covered federal changes to the Deferred Action for Childhood Arrivals (DACA) program and the public charge rule from 2018 through 2022 [17–19]. Two federal programs that have been associated with stigma and challenges to obtain health care [20, 21]. The negative effects of changes to federal immigration policies may have been exacerbated by the narratives that criminalized immigrants in the 2016 election, which were associated with poor mental health among Latino adolescents [22, 23]. Prior to the 2016 election, there was also the enactment of exclusionary state policies, such as Arizona’s Senate Bill 1070 (SB 1070) in 2010, which negatively impacted benefit utilization among immigrant women in Arizona before the policy went into effect [24]. Assessing the extent that news sources (i.e. newspapers) cover immigration policies offers an approach to measure the mechanisms by which policies may influence health behaviors.

### News Coverage as a Social Constructor of Immigration Policy

We argue that news coverage of immigration policy can generate distrust in health information and that this is an example of a mechanism through which immigration policy affects health. Health information that comes from the government may be a specific source for which there is distrust because the government is also the source of immigration policy. We operationalize news coverage as a “social constructor” of immigration policy and assess its relationship with distrust in health information from the government [25]. Social construction theory makes meaning of policy content and the social impact of policy on a target population group [25]. News coverage of immigration policies can influence local politics[26] and can stigmatize immigrants [27–29]. In a similar way, we can assess the relationship between distrust in health information and news coverage to understand the “meaning-making” and health impacts of immigration topics. For example, policy makers’ rhetoric and exclusive immigration policies may generate negative social constructions of immigrants and then be reinforced in news coverage; or vice versa. News environments also portray the target population of a new law or policy, such as Latinos or immigrants, as either threatening or beneficial [30, 31]. News coverage shapes the information environment, bringing attention to ideas, topics, and attitudes. Major newspaper stories are indicators of the broader information environment as they often create the information agenda by what is covered and how it is covered including in non-English and ethnic news media [32, 33]. More locally, newspapers are highly valued and a central fixture in shaping local attitudes [34]. Therefore, we conceptualize news coverage, which is primarily in English, as an indicator of the overall environment that is made up of different policies and topics impacting immigrants.

The news coverage about a set of immigration policies may influence the perception of the immigrant population group associated with those policies, and immigrants may also shift their views on and trust of policy makers or the government. For example, policies on immigrant policing resulted in decreased trust in information from the government among Latinos [2]. The influence of news coverage is of interest to public health, as the portrayal of a policy or its target population can shape how others interact with that population or shape the way the target population interacts with health care and public health agencies [35].

State policy climates simultaneously reflect both inclusionary and exclusionary priorities by state policy makers [27]. For example, among states with a high number of inclusive policies, media environments can have low coverage of inclusive policies, while having substantial coverage of exclusive policies [4]. Therefore, the type and intensity of news coverage are important elements to examining news as a social constructor of immigration policy climates. Additionally, examining these elements at a state level could also inform how Latino and immigrant health are shaped when state or federal immigration policies are out of alignment.

### Immigration Policy News Coverage and Distrust

Immigrant health researchers cite that there is a growing distrust in the government among Latinos [36]. Immigration policy news coverage may influence the perceived trustworthiness of government institutions in the same way that immigration policies and discrimination within health care settings create distrust among immigrants [37–39]. Distrust in health information may lead to decreased use of the few health benefits accessible to immigrants, similar to how immigrants’ distrust of using public benefits was associated with the public charge rule [21, 40].

In this study we examined the relationship between news coverage of immigration policy and distrust in health information and how this relationship may differ for Latinos and other racial/ethnic groups. We first sought to identify if Latinos reported high distrust in health information compared to other racial/ethnic groups, hypothesizing that Latinos would have similar levels of distrust in health information from the government compared to Black respondents and less distrust compared to White respondents due to the increased discrimination among minority racial/ethnic communities [29, 41]. We then assessed if news coverage in state capital newspapers of inclusive immigration policies and of exclusive immigration policies were associated with distrust in health information.

**Fig. 1:**
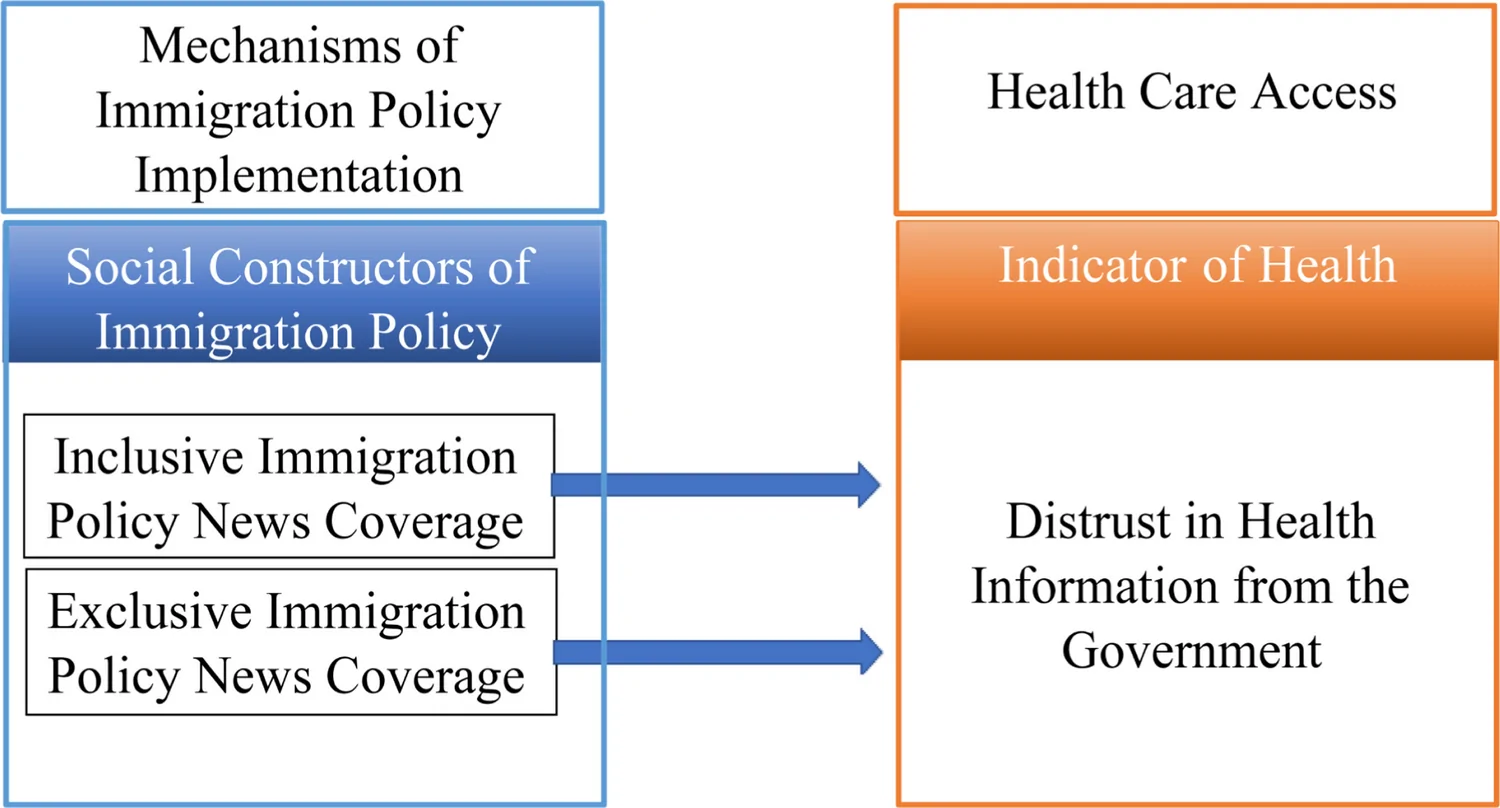
The relationship between mechanisms of immigration policy implementation and health care access

We expected inclusive policy news coverage would have a protective effect and would *not* be associated with high distrust in health information from the government, while exclusive policy news coverage would be associated with high distrust. Finally, we assessed differences in the relationship between news coverage and distrust between Latinos compared to other racial/ethnic groups, hypothesizing that race/ethnicity would modify the relationship. The conceptualization of using inclusive and exclusive immigration policy news coverage as social constructors of immigration policy and distrust in health information as an indicator of health is shown in Fig. 1.

Figure 1 shows the conceptual framework used to examine the relationship between immigration news coverage and distrust in health information from the government to understand the mechanisms of immigration policy implementation on health care access.

## Material and Methods

### Sample and Data Collection

This cross-sectional study combined three sources of data from 2010 through 2019: (i) an individual-level population data set from the National Cancer Institute’s Health Information National Trends Survey (HINTS); and (ii and iii) two state-level data sets, including a news coverage data set and a state policy index data set. We selected a subset of HINTS data guided by the time periods used in the news coverage data set [4]. Federal information processing system (FIPS) state codes matched each HINTS respondent’s state of residence with the state-level data sets. The final sample was 7,102 HINTS respondents.

#### HINTS Data

The individual-level data came from four cycles of pooled HINTS data. The pooled data included responses to HINTS 4 Cycle 1 (2011–2012), HINTS 4 Cycle 3 (2013), HINTS 5 Cycle 1 (2017), and HINTS 5 Cycle 3 (2019) [42–45]. Each HINTS cycle was a nationally representative survey of the non-institutionalized US population who were 18 or more years old. The full sampling methodology for these cycles is available online [42–45]. The four cycles of data were combined, as they used the same trust in health information questions.

#### News Coverage Data

The news coverage data set was a quantitative content analysis of newspaper articles from 15 US states that measured state-level coverage of inclusive immigration policies and exclusive immigration policies from 2010–2013 and 2017–2019, years selected to capture policy making during the Obama and Trump presidencies [4]. Newspapers are an indicator of the local news information climate, as they set the agenda in local media that is subsequently disseminated through other sources, such as television, radio, or social media, and in different languages [32–34]. The 15 states in the analysis were Arizona, California, Colorado, Florida, Georgia, Illinois, Maryland, Minnesota, New Jersey, New York, North Carolina, Oregon, Rhode Island, Texas, and Utah. These 15 states were selected because they had at least a 5% foreign-born population and their state capital newspapers had at least a 100 articles related to immigration policies in each time period [4]. Two measures for type of immigration policy news coverage were created from codes that captured the type of immigrant policies identified. Inclusionary news coverage was measured by counting newspaper articles covering integrative immigration policies such as the expansion of health care, safety net benefits, and employment protections. Exclusionary news coverage was measured by counting newspaper articles covering restrictive immigration policies and activities such as immigration enforcement.

#### State Characteristics Data Sets

We included data with policy and demographic characteristics for the state. First, we included an existing state policy index data set of state-level immigration policies [27]. This data set included a continuous measure for the number of inclusive state immigration policies and a continuous measure for the number of exclusive state immigration policies related to migration and immigrant rights in a state that were enacted by December 31, 2013 [27]. These policies included, for example, the presence of a law that facilitates a driver’s license for immigrants, expansion of Medicaid to undocumented pregnant women and children. Second, we included state-level population demographics obtained from the 2015 American Community Survey (ACS) by the US Census Bureau. The measures from this data set included the percent of Latino population in a state and the percent of the foreign-born population in a state.

### Measures

The dependent variable and individual level covariates were from HINTS. The independent variables are from the news coverage data set. The state-level covariates were from immigration policy context and state demographic data sets.

#### Dependent Variable

The outcome of interest was distrust in health information from the government. Participants were asked, “In general, how much would you trust information about health or medical topics from each of the following?” Response options included: Not at all, A little, Some, and A lot. We categorized ‘Not at all’ as a high distrust, and ‘A little’, ‘Some’, and ‘A lot’ as low distrust to create a dichotomous measure for distrust.

#### Independent Variables

We had two independent variables, inclusive and exclusive immigration policy news coverage. Inclusive news coverage was the percent of all articles that cover inclusive immigration policies in each state and ranged from 3 to 52%. Similarly, exclusive news coverage was the percent of all articles that cover exclusive immigration policies in each state and ranged from 25 to 85%. For ease of interpretation in the regression models, we converted the variables into measures in which each unit represents a 10% increase in coverage by multiplying each index measure by 10.

#### Covariates

##### Individual-Level Covariates

The individual-level covariates include race/ethnicity (Latino, Black, White (ref)). The Latino category included all respondents who identified as ‘Hispanic,’ regardless of self-identified race; while the White and Black categories included all respondents who identified as ‘Non-Hispanic.’ The other covariates included age (continuous), gender (female, male (ref)), education level (high school or more, less than high school (ref)), survey language (Spanish, English (ref)), rural designation (rural non-metropolitan, urban non-metropolitan, urban metropolitan (ref)), self-reported health (fair/poor, good, very good, or excellent (ref)), and survey year (2019, 2017, 2016, 2011–2012(ref)). These were used to control individual-level demographics that would have been associated with variations in distrust in health information. For example, race/ethnicity accounts for differences in newspaper reading behaviors.

##### State-Level Covariates

The state-level covariates include the inclusive immigration policy score (range: 0–12), the exclusive immigration policy score (range: 0–7). We used the measure of inclusive policies in each state as a proxy for the political context in which inclusive news coverage shaped immigration policy narratives or perceptions. We used the measure of exclusive policies as a proxy for the political context in which exclusive news coverage shaped immigration policy narratives or perceptions. We also used the percent of the state population that is noncitizen (range: 0%−100%) and percent of the state population that is Latino (range: 0%−100%). These were used to control for state-level population demographics that would have influenced social climates on immigration policy.

### Statistical Analysis

We calculated summary statistics to look at sample characteristics overall and by distrust in the government. Mixed-effects unweighted logistic regression models were used to estimate the relationship between each type of news coverage and distrust. Mixed-effects models allowed us to account for the nested effects of individuals within a state and examine the relationship between individual and state-level predictors. First, we tested the relationship between inclusive immigration policy news coverage and high distrust in health information from the government for the total sample, controlling for all covariates. Then using the same model, we tested the relationship between exclusive immigration policy news coverage and high distrust in health information, controlling for all covariates. We tested the same model with each type of news coverage separately, while controlling for both types of policy environments, to test the effects of news coverage alone on distrust.

We tested mixed effects logistic regression models with race/ethnicity and news coverage interaction terms to determine if the relationship between news coverage and high distrust was different for Latinos than for other race/ethnic groups. All statistical analyses were conducted using STATA 17.0 (StataCorp LP, College Station, TX).

## Results

Descriptive results showed the sample was predominantly White, identified as female, had more than a high school education, took the survey in English, reported very good health, and lived in metropolitan urban areas (see Table 1). 75% of respondents reported high distrust in health information from the government. Among those who identified as Latino, 74% reported high distrust compared to 77% of White respondents and 69% of Black respondents (see supplemental table A).

**Table 1:** Individual characteristics of full sample and by levels of distrust in health information from the government

	Full sample	Distrust in health information from the government	
	All (*n* = 7,102)	Low (*n* = 1,772)	High (*n* = 5,330)
	% or mean (sd)	% or mean (sd)	% or mean (sd)
**Total**	100.0	25.0	75.1
**Race/Ethnicity**			
Latino	22.0	23.3	21.6
White	63.5	58.6	65.1
Black	14.5	18.1	13.3
**Age (years)**	54.3 (16.5)	51.1 (16.5)	55.4 (16.3)
**Sex**			
Male	41.1	38.4	42.0
Female	58.9	61.6	58.0
**Education Level**			
Less than High School	7.0	6.2	7.2
High School or More	93.0	93.8	92.8
**Survey Language**			
English	97.6	97.3	97.7
Spanish	2.4	2.7	2.3
**Self-Reported Health**			
Excellent	12.9	15.6	12.0
Very Good	36.8	38.5	36.2
Good	35.1	32.2	36.0
Fair/Poor	15.3	13.7	15.8
**Rural Designation**			
Metropolitan—Urban	91.1	93.4	90.3
Non-Metropolitan—Urban	8.2	5.9	9.0
Non-Metropolitan—Rural	0.7	0.7	0.7
**Survey Year**			
2011–2012	25.7	25.7	25.7
2013	19.1	20.0	18.7
2017	20.6	23.7	19.6
2019	34.7	30.5	36.0

Source: HINTS Study 2011–2013, 2017, and 2019

Abbreviations: *sd* standard deviation

On average, 20% of states’ immigration policy coverage was of inclusive policies (see supplemental table B), while 50% of news coverage was of exclusive immigration policies.

The mixed-effects model assessing the relationship between inclusive news coverage and distrust showed that every ten percent increase in inclusive news coverage was associated with 10% higher odds of high distrust (OR: 1.10, 95% CI: 1.02–1.18), controlling for all covariates (see Table 2a). The odds of high distrust were 23% lower for Black respondents compared to Latino respondents (OR: 0.77, CI: 0.64–0.93). Reporting good or fair/poor health was associated with a 37% (OR: 1.37, CI:1.16–1.63) and 41% (OR:1.41, CI:1.15–1.74) higher odds of high distrust compared to reporting excellent health, respectively. Living in a non-metropolitan urban area, compared to a metropolitan urban area, was associated with 44% (OR:1.44, CI:1.15–1.81) higher odds of high distrust.

**Table 2:** Associations between immigration policy news coverage and high distrust in health information from the government

	**Model a**		**Model b**	
	OR	95% CI	OR	95% CI
**% Inclusive Immigration Policy News Coverage**	**1.10**	**(1.02, 1.18)**		
**% Exclusive Immigration Policy News Coverage**			**1.08**	**(1.03, 1.14)**
**Race/Ethnicity**				
Latino (ref)				
White	1.10	(0.94, 1.27)	1.10	(0.95, 1.27)
Black	**0.77**	**(0.64, 0.93)**	**0.78**	**(0.65, 0.94)**
**Age (years)**	**1.01**	**(1.01, 1.02)**	**1.01**	**(1.01, 1.02)**
**Sex**				
Male (ref)				
Female	0.91	(0.81, 1.01)	0.91	(0.81, 1.01)
**Education Level**				
Less than High School (ref)				
High School or More	0.93	(0.74, 1.18)	0.93	(0.73, 1.17)
**Survey Language**				
English (ref)				
Spanish	0.91	(0.63, 1.31)	0.90	(0.63,1.30)
**Self-Reported Health**				
Excellent (ref)				
Very Good	1.18	(1.00, 1.40)	1.18	(1.00, 1.40)
Good	**1.37**	**(1.16, 1.63)**	**1.38**	**(1.16, 1.64)**
Fair/Poor	**1.41**	**(1.15, 1.74)**	**1.41**	**(1.15, 1.74)**
**Rural Designation**				
Metropolitan—Urban (ref)				
Non-Metropolitan—Urban	**1.44**	**(1.15, 1.81)**	**1.46**	**(1.16, 1.83)**
Non-Metropolitan—Rural	1.00	(0.52, 1.94)	1.00	(0.52, 1.94)
**Survey Year**				
2011–2012 (ref)				
2013	0.94	(0.80, 1.11)	0.94	(0.80, 1.11)
2017	**0.80**	**(0.69, 0.94**	**0.81**	**(0.69, 0.94)**
2019	1.15	(0.99, 1.32)	1.15	(0.99, 1.33)
**% Latino**	1.00	(1.00, 1.01)	1.00	(1.00, 1.01)
**% Foreign Born**	0.99	(0.97, 1.00)	**0.98**	**(0.96, 1.00)**
**Inclusive Policy Score**	1.02	(0.99, 1.06)	1.01	(0.98, 1.05)
**Exclusive Policy Score**	0.99	(0.95, 1.04)	0.96	(0.92, 1.00)

Source: HINTS Study 2011–2013, 2017, and 2019; News coverage data set 2010–2013 and 2017–2019; State policy index data set 2013; ACS 2015

Abbreviations:* OR* Odds ratio, *CI* confidence interval

Bold font indicates statistical significance (95% CI excludes a coefficient of 1).

The mixed-effects model assessing the relationship between exclusive news coverage and distrust showed that every ten percent increase in exclusive news coverage was associated with 8% higher odds of high distrust (OR:1.08, 95% CI:1.03–1.14), controlling for all covariates (see Table 2b). Similar to inclusive news coverage, the odds of high distrust were 22% lower for Black respondents compared to Latino respondents (OR:0.78, CI:0.65–0.94); 38% and 41% higher for those with good (OR:1.38, 95% CI:1.16–1.64) and fair/poor (OR:1.41, 95% CI:1.15–1.74), compared to excellent health; and 46% higher for those living in a non-metropolitan compared to urban area (OR:1.46, 95% CI:1.16–1.83). The percent of foreign-born in a state was associated with a 2% lower odds of high distrust in the government for every increase in the percent of foreign-born residents in a state (OR:0.98, 95% CI:0.96–1.00).

When assessing if there were differences in the relationship between news coverage and distrust by race/ethnicity, the interaction terms were not statistically significant, suggesting that race/ethnicity did not moderate the associations between news coverage and distrust (see supplemental table C).

## Discussion

Our study aimed to examine news coverage of immigration policy as a mechanism by which immigration policy shapes distrust. Specifically, we tested the relationship between news coverage, as a “social constructor” of the immigration policy environment, and distrust in health information from the government. We used state-level measures for immigration policy news coverage and individual population-based data on distrust in health information from the government to conduct a multi-level analysis that controlled for state immigrant policy climates. Our study findings showed that news coverage of both inclusionary and exclusionary immigration policies was associated with a greater likelihood of high distrust in health information from the government. Overall, our findings suggest distrust is associated with *any* news coverage of immigration policy and may have a similar influence for Latino, White, and Black populations.

First, the association between inclusive news coverage and high distrust shows inclusive policies may have a similar influence as exclusionary immigration policies. This finding is contrary to our hypothesis that inclusive coverage may actually reduce distrust given the evidence that inclusive policies have been associated with better outcomes, such as health care access and utilization [5, 39]. This finding may be attributed to the salience of immigration in the news. Immigration is a topic that receives continuous coverage. For example, during the first months of the Biden administration, immigration was one of the top five covered policy topics across all major news sources [46]. Therefore, the salience of immigration as a *topic*, rather than a *type* of news coverage may have a role in shaping how populations are influenced by immigration policy. These findings also suggest that news coverage of immigration policy may have a role independent of policy in shaping health. By using news coverage as a social constructor of how individuals make meaning of immigration policies and how their behaviors are shaped as the targets of immigration policies, our findings capture how inclusive policy content or positive immigration rhetoric are insufficient to mitigate distrust. Through this study we add to growing literature that seeks to identify measures for inclusionary policies and show not all inclusive language or policies may have a protective influence on health [5, 47].

Second, our findings support existing evidence that exclusive immigration policies are associated with distrust in government among non-immigrant groups [48]. By using exclusionary news coverage for multiple states and using a population level outcome on distrust, we show the possible consequences of anti-immigrant rhetoric across various US states and racial/ethnic groups. Our study builds on immigrant health research that uses localized measures to examine and document the role of the social climates surrounding policies that target immigrants, but that may also impact population health was a whole. By analyzing distrust and state-level news coverage, we provide an idea of how different immigration climates potentially shape distrust in health information due to differences in the type of policies across the federal, state, and local level that can spillover across space and time through news communications [10, 36, 47, 49].

Third, our findings are important as we seek to further understand the local contexts and environments of Latino and immigrant reception that may shape their health. We anticipated Latinos would experience immigration policy news coverage differently than other racial/ethnic groups due to their long-standing legal status exclusions and marginalized racial identity [50]. However, the lack of significant findings in the interaction between race/ethnicity and news coverage suggests immigration policy news coverage has similar effects on different racial/ethnic groups when it comes to distrust. This aligns with past results showing Latinos, and Black respondents are both affected by discriminatory language, such as anti-immigrant narratives, and exclusionary policies [51, 52]. The relationship between news coverage and high distrust points to the social and political circumstances surrounding immigration and associated deteriorations in trust in the government, generally [48, 53]. By finding that distrust may not vary across racial/ethnic groups in relation to immigration policy, we contribute a counterpoint to suggestions that the trust of White population groups’ is primarily impacted [48, 53]. Past studies have also highlighted a growing distrust among immigrant and Latino population groups due to waves of anti-immigrant political messaging on immigration [54]. Data on trust across other national surveys show that there were similar levels of high distrust in the government at the time when our data was collected [55]. Since we also controlled for survey year, our findings on the association between inclusive policies and distrust, and the lack of variation between races/ethnicities, may also signal to some general cynicism around immigration as a topic across all racial/ethnic groups.

Notably, our study informs how trust in the government is shaped by state immigration policy climates and not just individual-level factors that have been previously assessed in research using HINTS. The limited evaluations of distrust with individual-level factors, such as cancer outcomes or where people seek information, are primarily attributed to the focus of the survey to optimize health information delivery [56–58]. However, by expanding the use of national survey data for trust in health information as an outcome and analyzing the relationship with measures for immigration policy climates, we can obtain a more comprehensive picture of the social and structural factors that shape population health. Distrust in health information is also an indicator of health or health care access [59, 60]. Therefore, in addition to capturing the social constructions of immigration policy through news coverage, we also add evidence on the influence of discriminatory rhetoric that may contextualize individual drivers of immigrant distrust within health care and government settings, and health care access, such as interpersonal discrimination.

### Limitations

Our study comes with some limitations. First, we use cross-sectional data and are not able to establish a causal relationship between news coverage and high distrust. However, by looking at the association between news coverage and distrust, while accounting for state-level variation, we elucidate how the social contexts surrounding immigration policy shape distrust.

Second, the timing of the outcome and the independent measures cannot be disentangled. The analysis does not represent data that was directly paired across time. The new coverage dataset is from 2010–2013 and 2017–2019, while the HINTS data is from 2011–2012, 2013, 2017, and 2019. However, as news coverage and discussions can often linger past a moment in time, we show a cumulative effect of inclusive and exclusive news coverage over the timeframe of our data.

Third, our news coverage measure used English language newspaper articles. While the articles used to construct these measures likely captured the full scope of immigration news during the study timeframe, due to the growing use of electronic media, our measures may have missed some articles or reporting on immigration policies that did not make it to newspaper articles. Future analyses could include measures reflecting additional electronic sources of news coverage, such as online, social media, or television reports.

Lastly, the HINTS data does not include immigration or documentation status information. HINTS collects information on whether participants are born in the United States, which is often used by immigration scholars to identify those who previously migrated. However, nationality data was not complete for all the pooled survey cycles. Our analysis also did not include Asian or Asian sub-ethnic population groups due to small sample size in our pooled data sets. Future analyses should include Asian population groups, as they are the fastest growing immigrant racial group in the United States [61] and have a long history of legal exclusion and experience ongoing discrimination in the United States [62–64].

### Future Research

Despite these limitations, the strengths of our study create opportunities for future research related to further assessing local measures, rhetoric and messaging in news coverage of immigration policies, and assessing the relationship between type of news coverage and trust in other sources of health information.

Our state-level analysis offers a robust assessment of the varied immigration policy climates across the United States. However, previous research has shown variation in perceptions of immigration across localized spaces such as border counties [65]. Future analyses can continue to look at the nested effects of state-level news coverage by assessing the relationship of news coverage and distrust by counties. A county-level nested analysis can account for differences in distrust by counties with high anti-immigrant attitudes compared to those with more inclusive attitudes, such as sanctuary policies, and assess changes to the association between distrust and news coverage.

Additionally, the specific messages or patterns by which inclusive immigration policy messages in the news are associated with high distrust are worthy of further inquiry as inclusive messages intended to promote policies of immigrant inclusion may instead further erode perceptions of the government due to the topic of immigration alone. This could include using measures that capture the intensity of the coverage at a granular level across time of an important inclusive policy or event, or by different types of inclusive messages. This may require also assessing news sources in different languages as Spanish print media frames news comparing Latino people vs non-Latinos, whereas English print media frames news comparing White people vs Black people or other population groups [33]. Our study only reflects popular articles in English and controlling for race/ethnicity in our main effects model accounts for differences in newspaper reading behaviors.

Future analyses can look at distrust across additional health information sources who are more trustworthy for immigrant population groups to assess if the organization type mitigates distrust despite the political climate. We also acknowledge governmental entities, such as public health agencies, are sources of health-related information for Latinos and immigrants enrolled in government safety net programs. However, local community-based or community health organizations, are often the trusted messengers among non-white racial/ethnic groups [66]. We help inform the contexts in which trusted community-based organizations operate as they are often cited as a solution to communicating information in anti-immigrant policy climates [67].

## Conclusion

Assessing immigration policy news coverage as a policy-related factor that influences distrust in health information is an approach to understand how immigration policy shapes immigrant health. By finding inclusive and exclusive immigration policy news coverage are both associated with high distrust in health information from the government, we expand our understanding of the mechanisms by which social climates surrounding immigration policy and immigration rhetoric shape immigrant health. Our findings suggest the framing, attitudes, and narratives used in news coverage around immigration as a news topic alone may have a similar role in influencing health as do different types of immigration policies. Ultimately, this study adds further evidence to the role social climates and attitudes towards immigrants have on their health in the absence of immigration reform.

## Supplementary Information

Below is the link to the electronic supplementary material.

Supplementary file1 (DOCX 31.2 KB)
