# Supplementary material for: Immigration Policies Shape Latinos’ Trust in Health Information
Source: J Racial Ethn Health Disparities. 2025 Jul 23;13(5):4162–73. doi: 10.1007/s40615-025-02566-8 (PMC13546157; doi:10.1007/s40615-025-02566-8)
Supplement: Supplementary file 1 [file 40615_2025_2566_MOESM1_ESM.docx]

**Supplemental Tables**

Supplemental Table A. Bivariate statistics for individual demographics by levels distrust in health information from the government, 2011-13, 2017, and 2019

|  | **Low distrust** | **High distrust** |
| --- | --- | --- |
|  | % or mean (sd) | % or mean (sd) |
| **% of sample** | 25.0 | 75.1 |
| **Race/Ethnicity** |  |  |
| Latino | **26.3** | **73.7** |
| White | **23.1** | **76.9** |
| Black | **31.1** | **68.9** |
| **Age (years)** | **51.11 (16.47)** | **55.37 (16.30)** |
| **Sex** |  |  |
| Male | **23.3** | **76.7** |
| Female | **26.1** | **73.9** |
| **Education Level** |  |  |
| Less than High School | 22.3 | 77.7 |
| High School or More | 25.2 | 74.9 |
| **Survey Language** |  |  |
| English | 24.9 | 75.12 |
| Spanish | 27.8 | 72.25 |
| **Self-Reported Health** |  |  |
| Excellent | **30.2** | **69.8** |
| Very Good | **26.1** | **73.9** |
| Good | **22.9** | **77.1** |
| Fair/Poor | **22.4** | **77.6** |
| **Rural Designation** |  |  |
| Metropolitan - Urban | **25.6** | **74.4** |
| Non-Metropolitan - Urban | **18.0** | **82.0** |
| Non-Metropolitan - Rural | **23.5** | **76.5** |
| **Survey Year** |  |  |
| 2011-2012 | **25.0** | **75.0** |
| 2013 | **26.2** | **73.8** |
| 2017 | **28.7** | **71.3** |
| 2019 | **22.0** | **78.0** |
| Total (n) | **1,772** | **5,330** |
| Source: HINTS Study 2011-2013, 2017, and 2019  Abbreviations: sd = standard deviation  Bold font indicates statistical significance (95% CI excludes a coefficient of 1) for categorical differences. | | |

Supplemental Table B. State Characteristics and % with high distrust in health information from the government across all 15 sample states, 2010-2013, 2017-2019

| **State** | **n = 7,102** | **% of sample** | **% of sample with high distrust*** | **% with high distrust in state** | **% Inclusive Immigration Policy News Coverage*** | **% Exclusive Immigration Policy News Coverage*** | **% Latino*** | **% Foreign Born*** | **Inclusive Immigration Policy Score*** | **Exclusive Immigration Policy Score*** |
| --- | --- | --- | --- | --- | --- | --- | --- | --- | --- | --- |
| **Arizona** | 285 | 4 | 4 | 80 | 52 | 85 | 31 | 14 | 5 | 5 |
| **California** | 1444 | 20 | 21 | 76 | 28 | 57 | 39 | 27 | 11 | 1 |
| **Colorado** | 222 | 3 | 3 | 78 | 15 | 44 | 21 | 10 | 7 | 2 |
| **Florida** | 878 | 12 | 12 | 75 | 14 | 58 | 24 | 20 | 6 | 5 |
| **Georgia** | 444 | 6 | 6 | 76 | 32 | 50 | 9 | 10 | 2 | 4 |
| **Illinois** | 466 | 7 | 6 | 74 | 11 | 25 | 17 | 14 | 7 | 1 |
| **Maryland** | 269 | 4 | 4 | 70 | 14 | 44 | 9 | 15 | 6 | 3 |
| **Minnesota** | 202 | 3 | 3 | 82 | 22 | 63 | 5 | 8 | 8 | 4 |
| **New Jersey** | 306 | 4 | 4 | 71 | 14 | 41 | 19 | 22 | 5 | 4 |
| **New York** | 669 | 9 | 9 | 72 | 3 | 39 | 19 | 23 | 12 | 4 |
| **North Carolina** | 390 | 5 | 5 | 74 | 21 | 38 | 9 | 8 | 3 | 5 |
| **Oregon** | 116 | 2 | 2 | 78 | 17 | 32 | 13 | 10 | 8 | 3 |
| **Rhode Island** | 27 | 0 | 0 | 81 | 30 | 56 | 14 | 13 | 6 | 3 |
| **Texas** | 1296 | 18 | 18 | 75 | 18 | 45 | 39 | 17 | 7 | 5 |
| **Utah** | 88 | 1 | 1 | 73 | 16 | 34 | 14 | 8 | 4 | 3 |
| **HINTS Sample Average** | 7102 | 100 | 75 | - | 20 | 49 | 25 | 18 | 7.43 (2.92) | 3.46 (1.65) |
| **Source:** HINTS 2011-2013, 2017, and 2019; Immigration news coverage data set, 2010-2013, 2017-2019; State Policy Index data set 2013; ACS 2015  * indicates statistical significance (95% CI excludes a coefficient of 1) for state-level group differences. | | | | | | | | | | |

Supplemental Table C. Associations between (1) types of immigration policy news coverage and (2) the interaction with race/ethnicity

|  | **Inclusive new coverage** | | | | **Exclusive news coverage** | | | |
| --- | --- | --- | --- | --- | --- | --- | --- | --- |
|  | **Model 1a** | | **Model 2a** | | **Model 1b** | | **Model 2b** | |
|  | **OR** | **95% CI** | **OR** | **95% CI** | **OR** | **95% CI** | **OR** | **95% CI** |
| **% Inclusive Immigration Policy News Coverage** | **1.10** | **(1.02, 1.18)** | 1.07 | (0.95, 1.21) | **-** | **-** | **-** | **-** |
| **% Exclusive Immigration Policy News Coverage** | **-** | **-** | **-** | **-** | **1.08** | **(1.03, 1.14)** | **1.14** | **(1.02, 1.27)** |
| **Race/Ethnicity** |  |  |  |  |  |  |  |  |
| Latino (ref) |  |  |  |  |  |  |  |  |
| White | 1.11 | (0.96, 1.28) | 0.99 | (0.73, 1.36) | 1.11 | (0.96, 1.28) | 1.47 | (0.79, 2.74) |
| Black | **0.78** | **(0.65, 0.93)** | 0.85 | (0.57, 1.27) | **0.79** | **(0.66, 0.95)** | 1.16 | (0.50, 2.67) |
| **Age (years)** | **1.01** | **(1.01, 1.02)** | **1.01** | **(1.01, 1.02)** | **1.01** | **(0.66, 1.02)** | **1.01** | **(1.01, 1.02)** |
| **Sex** |  |  |  |  |  |  |  |  |
| Male (ref) |  |  |  |  |  |  |  |  |
| Female | 0.91 | (0.81, 1.01) | 0.91 | (0.81, 1.01) | 0.91 | (0.81, 1.01) | 0.91 | (0.81, 1.02) |
| **Education Level** |  |  |  |  |  |  |  |  |
| Less than High School (ref) |  |  |  |  |  |  |  |  |
| High School or More | 0.94 | (0.75, 1.19) | 0.93 | (0.74, 1.18) | 0.93 | (0.74, 1.18) | 0.92 | (0.73, 1.16) |
| **Survey Language** |  |  |  |  |  |  |  |  |
| English (ref) |  |  |  |  |  |  |  |  |
| Spanish | 0.91 | (0.63, 1.31) | 0.90 | (0.63, 1.30) | 0.90 | (0.63, 1.30) | 0.90 | (0.63, 1.30) |
| **Self-Reported Health** |  |  |  |  |  |  |  |  |
| Excellent (ref) |  |  |  |  |  |  |  |  |
| Very Good | 1.18 | (1.00, 1.40) | 1.18 | (1.00, 1.40) | 1.18 | (1.00, 1.40) | 1.19 | (1.00, 1.40) |
| Good | **1.37** | **(1.15, 1.63)** | **1.38** | **(1.16, 1.64)** | **1.38** | **(1.16,1.64)** | **1.38** | **(1.16, 1.64)** |
| Fair/Poor | **1.41** | **(1.14, 1.73)** | **1.41** | **(1.15, 1.74)** | **1.41** | **(1.14, 1.73)** | **1.41** | **(1.15, 1.74)** |
| **Rural Designation** |  |  |  |  |  |  |  |  |
| Metropolitan - Urban (ref) |  |  |  |  |  |  |  |  |
| Non-Metropolitan - Urban | **1.44** | **(1.15, 1.81)** | **1.45** | **(1.16, 1.82)** | **1.46** | **(1.16, 1.83)** | **1.46** | **(1.16, 1.83)** |
| Non-Metropolitan - Rural | 1.00 | (0.52, 1.94) | 0.99 | (0.51, 1.93) | 1.00 | (0.52, 1.94) | 1.00 | (0.52, 1.94) |
| **Survey Year** |  |  |  |  |  |  |  |  |
| 2011-2012 (ref) |  |  |  |  |  |  |  |  |
| 2013 | 0.94 | (0.80, 1.11) | 0.94 | (0.80, 1.11) | 0.94 | (0.80, 1.11) | 0.94 | (0.80, 1.11) |
| 2017 | **0.80** | **(0.69, 0.94)** | **0.80** | **(0.69, 0.94)** | **0.81** | **(0.69, 0.95)** | **0.80** | **(0.69, 0.94)** |
| 2019 | 1.15 | (0.99, 1.33) | 1.15 | (0.99, 1.32) | 1.15 | (0.99, 1.33) | 1.15 | (0.99, 1.33) |
| **% Latino** | 1.00 | (1.00, 1.01) | 1.00 | (1.00, 1.01) | 1.00 | (1.00, 1.01) | 1.00 | (1.00, 1.01) |
| **% Foreign Born** | 0.99 | (0.97, 1.00) | 0.99 | (0.97, 1.00) | **0.98** | **(0.96, 1.00)** | **0.98** | **(0.96, 1.00)** |
| **Inclusive Policy Score** | 1.02 | (0.99, 1.06) | 1.02 | (0.99, 1.06) | 1.01 | (0.98, 1.05) | 1.01 | (0.98, 1.05) |
| **Exclusive Policy Score** | 0.99 | (0.95, 1.04) | 0.99 | (0.95, 1.04) | 0.96 | (0.92, 1.00) | 0.96 | (0.93, 1.00) |
| **Interaction with Race/Ethnicity** |  |  |  |  |  |  |  |  |
| Latino (ref) |  |  |  |  |  |  |  |  |
| White | **-** | **-** | 1.61 | (0.41, 6.29) | **-** | **-** | 0.57 | (0.17, 1.91) |
| Black | **-** | **-** | 0.55 | (0.08, 3.65) | **-** | **-** | 0.45 | (0.08, 2.54) |
| Source: HINTS Study 2011-2013, 2017, and 2019; News coverage dataset 2010-2013 and 2017-2019; State policy index data set 2013; ACS 2015  Abbreviations: sd = standard deviation. OR = odds ratio.  Bold font indicates statistical significance (95% CI excludes a coefficient of 1). | | | | | | | | |
